# Supplementary material for: Causal Relationship between Aging and Anorexia Nervosa: A White-Matter-Microstructure-Mediated Mendelian Randomization Analysis
Source: Biomedicines. 2024 Aug 16;12(8):1874. doi: 10.3390/biomedicines12081874 (PMC11351342; doi:10.3390/biomedicines12081874)
Supplement: Supplementary file 1 [file biomedicines-12-01874-s001.zip › Supplementary Figure/Figure S4/Singe SNP plot of MR analysis for TL to ubm-b-1500.pdf]

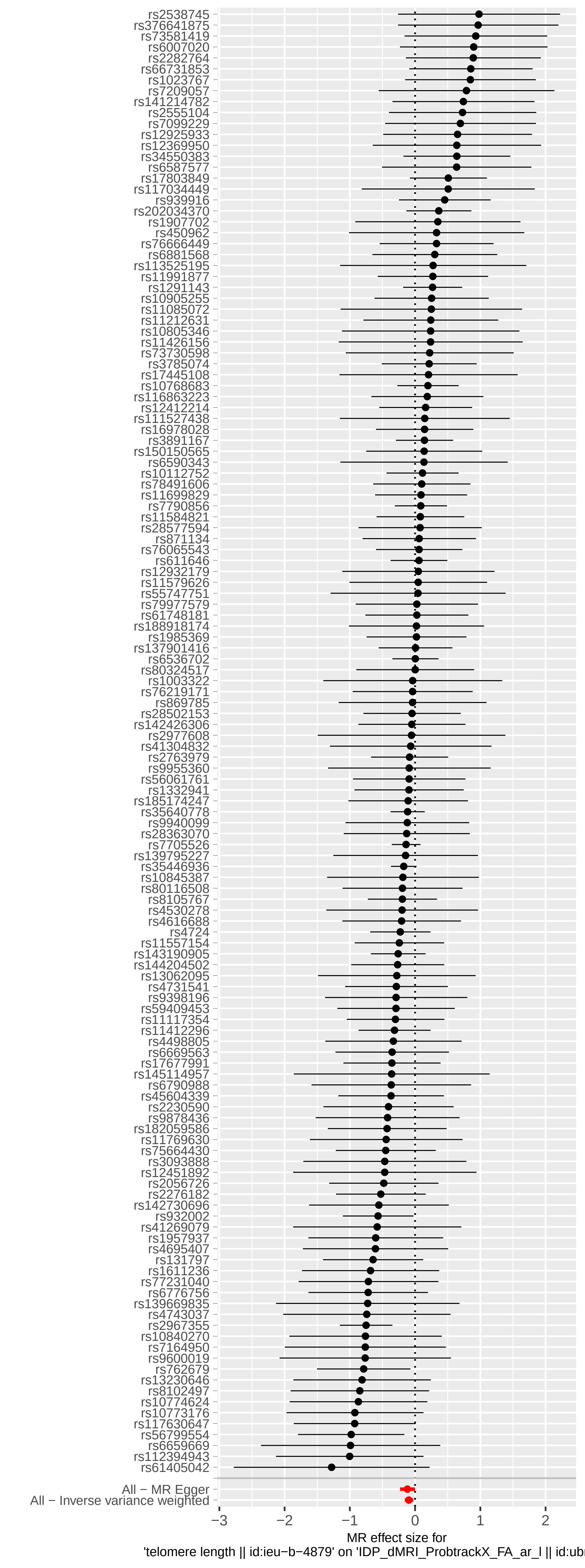
rs2538745
rs376641875
rs73581419
rs6007020
rs2282764
rs66731853
rs1023767
rs7209057
rs141214782
rs2555104
rs7099229
rs12925933
rs12369950
rs34550383
rs6587577
rs17803849
rs117034449
rs939916
rs202034370
rs1907702
rs450962
rs76666449
rs6881568
rs113525195
rs11991877
rs1291143
rs10905255
rs11085072
rs11212631
rs10805346
rs11426156
rs73730598
rs3785074
rs17445108
rs10768683
rs116863223
rs12412214
rs111527438
rs16978028
rs3891167
rs150150565
rs6590343
rs10112752
rs78491606
rs11699829
rs7790856
rs11584821
rs28577594
rs871134
rs76065543
rs611646
rs12932179
rs11579626
rs55747751
rs79977579
rs61748181
rs188918174
rs1985369
rs137901416
rs6536702
rs80324517
rs1003322
rs76219171
rs869785
rs28502153
rs142426306
rs2977608
rs41304832
rs2763979
rs9955360
rs56061761
rs1332941
rs185174247
rs35640778
rs9940099
rs28363070
rs7705526
rs139795227
rs35446936
rs10845387
rs80116508
rs8105767
rs4530278
rs4616688
rs4724
rs11557154
rs143190905
rs144204502
rs13062095
rs4731541
rs9398196
rs59409453
rs11117354
rs11412296
rs4498805
rs6669563
rs17677991
rs145114957
rs6790988
rs45604339
rs2230590
rs9878436
rs182059586
rs11769630
rs75664430
rs3093888
rs12451892
rs2056726
rs2276182
rs142730696
rs932002
rs41269079
rs1957937
rs4695407
rs131797
rs1611236
rs77231040
rs6776756
rs139669835
rs4743037
rs2967355
rs10840270
rs7164950
rs9600019
rs762679
rs13230646
rs8102497
rs10774624
rs10773176
rs117630647
rs56799554
rs6659669
rs112394943
rs61405042
All − MR Egger
All − Inverse variance weighted
−3
−2
−1
0
1
2
MR effect size for
'telomere length || id:ieu−b−4879' on 'IDP_dMRI_ProbtrackX_FA_ar_l || id:ubm
